# Mechanobiology of Microvascular Function and Structure in Health and Disease: Focus on the Coronary Circulation

**DOI:** 10.3389/fphys.2021.771960

**Published:** 2021-12-23

**Authors:** Maarten M. Brandt, Caroline Cheng, Daphne Merkus, Dirk J. Duncker, Oana Sorop

**Affiliations:** ^1^Division of Experimental Cardiology, Department of Cardiology, Erasmus MC, University Medical Center Rotterdam, Rotterdam, Netherlands; ^2^Division of Internal Medicine and Dermatology, Department of Nephrology and Hypertension, University Medical Center Utrecht, Utrecht, Netherlands; ^3^Walter Brendel Center of Experimental Medicine (WBex), LMU Munich, Munich, Germany; ^4^German Center for Cardiovascular Research (DZHK), Partner Site Munich, Munich Heart Alliance (MHA), Munich, Germany

**Keywords:** microvascular remodeling, microvascular density, microvascular dysfunction, coronary blood flow, endothelial dysfunction, ischemic heart disease, microvascular disease

## Abstract

The coronary microvasculature plays a key role in regulating the tight coupling between myocardial perfusion and myocardial oxygen demand across a wide range of cardiac activity. Short-term regulation of coronary blood flow in response to metabolic stimuli is achieved via adjustment of vascular diameter in different segments of the microvasculature in conjunction with mechanical forces eliciting myogenic and flow-mediated vasodilation. In contrast, chronic adjustments in flow regulation also involve microvascular structural modifications, termed remodeling. Vascular remodeling encompasses changes in microvascular diameter and/or density being largely modulated by mechanical forces acting on the endothelium and vascular smooth muscle cells. Whereas in recent years, substantial knowledge has been gathered regarding the molecular mechanisms controlling microvascular tone and how these are altered in various diseases, the structural adaptations in response to pathologic situations are less well understood. In this article, we review the factors involved in coronary microvascular functional and structural alterations in obstructive and non-obstructive coronary artery disease and the molecular mechanisms involved therein with a focus on mechanobiology. Cardiovascular risk factors including metabolic dysregulation, hypercholesterolemia, hypertension and aging have been shown to induce microvascular (endothelial) dysfunction and vascular remodeling. Additionally, alterations in biomechanical forces produced by a coronary artery stenosis are associated with microvascular functional and structural alterations. Future studies should be directed at further unraveling the mechanisms underlying the coronary microvascular functional and structural alterations in disease; a deeper understanding of these mechanisms is critical for the identification of potential new targets for the treatment of ischemic heart disease.

## Introduction

The coronary microvasculature plays a key role in the tight coupling between myocardial perfusion and myocardial oxygen demand across a wide range of cardiac activity. Short-term regulation of coronary blood flow (CBF) in response to metabolic stimuli is achieved via adjustment of vascular diameter in different segments of the microvasculature in conjunction with mechanical forces eliciting myogenic and flow-mediated responses (Duncker and Bache, 2008). In contrast, chronic adjustments in flow regulation also involve structural modifications of the microvasculature, termed remodeling. Vascular remodeling encompasses changes in microvascular diameter and/or density and is largely modulated by mechanical forces acting on the endothelium and vascular smooth muscle cells (VSMCs). Moreover, metabolic and endothelial factors controlling vascular tone also play an important role in maintaining the integrity of the microvascular network. Such factors have been shown to be altered in pathological situations.

Especially in the setting of ischemic heart disease (IHD), distal to a proximal epicardial artery stenosis, mechanical determinants of vascular tone, such as perfusion pressure, extravascular compression and flow, are altered, possibly contributing to microvascular remodeling. Moreover, even in the absence of a coronary obstruction, risk factors commonly seen in patients with IHD, such as diabetes, hypercholesterolemia, hypertension and aging, result in microvascular dysfunction and remodeling, impairing myocardial perfusion (Padro et al., 2020; Sorop et al., 2020). Such risk factors could also exacerbate microvascular structural and functional alterations in the myocardium distal to a flow-limiting coronary stenosis possibly contributing to the residual ischemia still present in many patients long after recanalization of the obstructed artery.

Although in recent years more data have been gathered regarding coronary microvascular function in patients at different stages of cardiovascular disease, the microvascular structural alterations, including vascular (arteriolar and capillary) density and collateralization, as well as remodeling of the vascular wall, still remain incompletely understood (Padro et al., 2020; Sorop et al., 2020). A deeper understanding of the mechanisms responsible for these changes is critical for the identification of potential new targets for the treatment of IHD. In this review, we present an overview of available data in humans and animal models, regarding the alterations in microvascular structure from an early stage, with the mere presence of cardiovascular risk factors, to a later stage, with overt IHD, with considerable hemodynamic consequences. Vascular function and its contribution to remodeling has been reviewed elsewhere (Pant et al., 2014; Fang et al., 2019). Here, we will mainly focus on the effects of biomechanical forces on vascular tone and structure in health and disease.

## Coronary Microvascular Function and Structure in the Healthy Heart

The primary function of the coronary circulation is to transport oxygen and nutrients to the myocardium. During increased metabolic demand, myocardial perfusion must increase commensurately with the increase in myocardial oxygen consumption, which is mainly achieved via regulation of coronary microvascular resistance (Hastings et al., 1982; Duncker and Bache, 2008; Goodwill et al., 2017). Several decades of intense scientific effort has improved our understanding of the physiological processes involved in these adaptive responses and the mechanisms involved. We will briefly discuss the different regulators of vascular tone and structure, focusing primarily on mechanical factors.

### Control of Vascular Tone in the Healthy Heart

The increase in myocardial oxygen consumption of the left ventricle, as required during exercise or stress, is principally met by an increase in oxygen delivery and thus in CBF, as the myocardium already has a high oxygen extraction at rest (>70%). The increase in CBF can amount up to 4–5 times the resting flow in the healthy heart (Duncker and Bache, 2008), and is mainly achieved by a reduction in vascular resistance. Under normal circumstances, proximal epicardial arteries (>400 μm in diameter) serve as conduit vessels as they contribute minimally (<5%) to overall coronary vascular resistance (Goodwill et al., 2017). The major loci of coronary vascular resistance are the coronary small arteries and arterioles (20–400 μm in diameter), responding to changes in physical forces (wall stress and shear stress), as well as metabolic needs of the tissue, while less than 20% of the resistance resides in the capillaries and venules (Chilian et al., 1986; Goodwill et al., 2017). The acute and/or chronic modulation of coronary vascular resistance in response to changes in myocardial oxygen demand, involves both active (vascular tone) and passive (extravascular compressive and intravascular distending mechanical forces) changes in vessel diameter.

**Figure 1:**
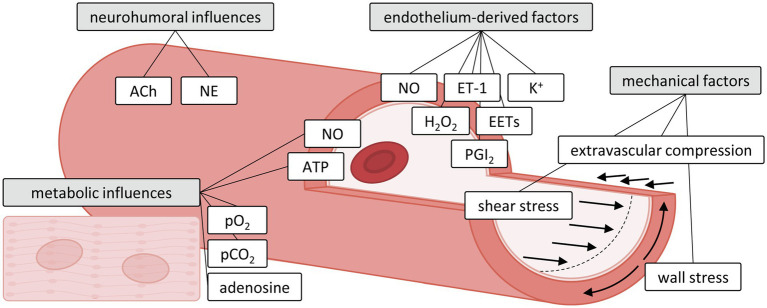
Influences of metabolic, neurohumoral, endothelium-derived and mechanical factors on the microvasculature. Adapted with permission from Duncker and Bache (2008). ACh, acetylcholine; ATP, adenosine triphosphate; EETs, epoxyeicosatrienoic acids; ET-1, endothelin-1; H_2_O_2_, hydrogen peroxide; NE, norepinephrine; NO, nitric oxide; pCO_2_, dissolved carbon dioxide; PGI2, prostacyclin; pO_2_, oxygen tension.

An optimal level of vascular diameter is thus achieved by a tightly regulated balance between a variety of vasoactive mechanisms, including metabolic, endothelial, neurohumoral and mechanical factors. The latter includes passive vascular responses to changes in the mechanical environment produced by myocardial compression and intravascular distending pressure, but also active responses contributing to vascular tone, such as myogenic response and flow-mediated response induced by changes in the perfusion pressure and shear stress. These mechanisms exert specific influences on different segments of the microvasculature (Jones et al., 1993a; Muller et al., 1996; Laughlin et al., 2012; Duncker et al., 2015) with the distal, smallest arterioles (<100 μm diameter) being most sensitive to myocyte-derived metabolic stimuli, whereas wall stress-induced myogenic mechanisms are dominant in the intermediate larger arterioles and small arteries (100–200 μm) and flow-mediated dilation dominates the vasomotor tone of small arteries (200–400 μm). The most important vasoactive mechanisms regulating vascular tone are individually described below and summarized in Figure 1.

#### Neurohumoral Factors

Neural stimulation also affects tone, as sympathetic and parasympathetic (vagal) nerves innervate different segments of the coronary vasculature, although their influence on coronary vascular tone differs between rest and exercise. Thus, while cardiac sympathetic activity is limited at rest, increased sympathetic activity contributes to exercise-induced hyperemia (Duncker and Bache, 2008; Goodwill et al., 2017). Neural stimulation exerts its effects on vascular tone via an interaction between the direct effects on vascular smooth muscle cells and stimulation of nitric oxide (NO) release from the endothelium.

During sympathetic activation, coronary tone is modulated by norepinephrine release from sympathetic nerves, and by circulating epinephrine and norepinephrine (Goodwill et al., 2017). In conduit arteries, sympathetic stimulation leads to a net vasodilator response mediated by an interaction between alpha_1_-mediated vasoconstriction and beta-mediated vasodilation (Goodwill et al., 2017). In the coronary resistance vessels, the effect of sympathetic activation on vascular tone depends on the net actions of beta_1_-mediated increases in myocardial oxygen consumption, beta_1_- and beta_2_-mediated coronary vasodilation, and alpha_1_-mediated vasoconstriction (Laughlin et al., 2012). In the healthy heart, exercise-induced beta-adrenergic “feed-forward” dilation predominates over alpha adrenergic constriction (Duncker and Bache, 2008), with contributions of both beta_2_- and beta_1_-receptors (Gao et al., 2010), resulting in an increase in CBF that matches the increase in myocardial oxygen consumption. Alpha-adrenergic blockade can induce vasodilation by blocking the vasoconstrictor influence of alpha_1_- and alpha_2_-adrenoceptors, with alpha_1_-adrenoceptors being more predominant in small coronary arteries (>100 μm) and both alpha_1_- and alpha_2_-adrenoceptors present in arterioles (<100 μm) (Duncker and Bache, 2008). Data show little evidence for alpha-adrenergic coronary vasoconstrictor influences at rest, but alpha-adrenergic constriction is augmented both at rest and during exercise in the presence of coronary endothelial dysfunction involving both alpha_1_- and alpha_2_-adrenoceptors (Heusch et al., 2000; Laughlin et al., 2012).

The role of vagal activity in the control of CBF at rest and during exercise has been shown to be negligible, as vagal tone to the myocardium is progressively withdrawn during increased levels of exercise (Duncker and Bache, 2008) although this appears to be species-dependent. Human and canine coronary resistance arteries have shown endothelium-dependent dilation to acetylcholine resulting in increases in CBF (Laughlin et al., 2012). Besides its role as a cholinergic neurotransmitter, non-neuronal acetylcholine was also shown to play a role in endothelial mechanotransduction, by being released by the endothelial cells (ECs) in response to flow, resulting in vasodilation (Wilson et al., 2016). Additionally, in swine, in which acetylcholine produces vasoconstriction, an interaction was observed of sympathetic and parasympathetic influences on coronary vascular tone during exercise. Thus, beta-adrenergic vasodilation was enhanced by withdrawal of the muscarinic receptor-mediated inhibition (Duncker et al., 1998). However, in dogs, where acetylcholine results in net vasodilation, parasympathetic effects are weak at rest and negligible during exercise (Duncker and Bache, 2008).

In conclusion, the autonomic nervous system is able to modulate the coupling between coronary flow and myocardial metabolism, with minimal activity at rest, and net beta-adrenergic feed-forward vasodilation during exercise.

#### Endothelial Factors

Vascular endothelium is one of the major determinants of vascular tone, by releasing various vasoactive substances in response to different stimuli. These factors include powerful vasodilators, such as NO, prostaglandins, and epoxyeicosatrienoic acids (EETs), K^+^ and H_2_O_2_, which induce endothelium-derived hyperpolarization (EDH; Laughlin et al., 2012). Studies have shown that NO-dependent responses occur primarily in small arteries and large arterioles (100–300 μm) and involve cyclic guanosine monophosphate (cGMP)-dependent hyperpolarization of VSMCs via the opening of specific K^+^ channels (Dick and Tune, 2010). Prostaglandin release has been shown to contribute to coronary reactive hyperemia but only in the presence of inhibition of NO synthesis, suggesting interaction of the two mechanisms (Puybasset et al., 1996). Additionally, although the exact nature of the factors involved in EDH (acting primarily on arterioles <100 μm) remains incompletely understood, several potential candidates (EETs, K^+^, H_2_O_2_) have been shown to be regulators of vascular tone in response to different stimuli such as shear stress, bradykinin or adenosine stimulation (Gutterman et al., 2016). Conversely, ET-1 has been identified as potent vasoconstrictor. Although its role appears to be rather modest under physiologic conditions, it becomes more important in disease states such as coronary artery disease (Sorop et al., 2008). Moreover, prostaglandin F2α, thromboxane and serotonin have been shown to be potent vasoconstrictors, also in pathological situations such as endothelial injury and coronary artery disease (Goodwill et al., 2017).

#### Metabolic Factors

Metabolic activity of the heart is one of the major factors regulating coronary vascular resistance. In order to maintain function and accommodate the high metabolic demand, the heart relies on aerobic metabolism to convert metabolic substrates into energy molecules, ATP. The exact nature of the factors and mechanisms responsible for local microvascular metabolic tone control is still not completely understood (Deussen et al., 2012), but several tissue-derived metabolites have traditionally been proposed to play a role in the regulation of coronary microvascular resistance during increased metabolic demand. These include dissolved O_2_ and CO_2_, as well as adenosine, involving activation of various K^+^ channels (Gerlach and Deuticke, 1966; Tune, 2007; Goodwill et al., 2017), although the specific contribution of each type of K^+^ channel remains a matter of debate. An increase in myocardial adenosine was originally proposed to link the flow regulation during changes in metabolism (Feigl, 2004). Thus, during increased myocardial oxygen consumption, a fall in myocardial oxygen tension could produce myocardial adenosine release and subsequent coronary vasodilation (Berne, 1963). However, in both human and animal studies, adenosine blockade did not affect vasodilation during physiological increases in myocardial oxygen consumption (Bache et al., 1988; Edlund et al., 1989; Duncker and Bache, 2008) in the healthy heart. In contrast, adenosine was shown to play an important role in coronary vasodilation during ischemia (Laxson et al., 1993). More recently, other factors, such as adenine nucleotides (ATP) or NO released from erythrocytes during hypoxia, have been proposed to mediate metabolic vasodilation (Gorman et al., 2010), although proof for their involvement is still lacking (Laughlin et al., 2012; Goodwill et al., 2017). $K_{\mathrm{ATP}}^{}$ channel blockade impaired CBF in hypoxia; however, this response was only transient and $K_{\mathrm{ATP}}^{}$ channel blockade did not impair exercise hyperemia (Duncker et al., 1993). Importantly, the mechanisms described above do not work independently, as studies in animal models have suggested that $K_{\mathrm{ATP}}^{}$ channels, adenosine and NO interact in different manners to control coronary flow control during exercise. Thus, in dogs these three interacting mechanisms fully control coronary perfusion during exercise (Ishibashi et al., 1998). This may at least in part be species dependent, as in swine a residual exercise-induced vasodilation upon inhibition of these mechanisms was still present (Merkus et al., 2003). Finally, mitochondria-derived hydrogen peroxide (H_2_O_2_) has also been suggested as possible mediator coupling CBF to metabolism in the heart, through modulating the opening probability of voltage-gated K^+^ channels. Consistent with this proposal, blockade of voltage-gated K^+^ channels was shown to impair the balance between CBF and myocardial metabolism (Berwick et al., 2012; Deussen et al., 2012; Goodwill et al., 2017; Ohanyan et al., 2017). Nevertheless, despite intense research efforts, the mechanisms controlling CBF during high metabolic demand still remain incompletely understood.

#### Mechanical Factors

In addition to the above described mechanisms, the vascular wall also contributes to modulation of vascular tone in response to biophysical forces exerted by both the flowing blood as well as the surrounding tissue. Flowing blood not only exerts a frictional force on the endothelial lining termed fluid shear stress, but the vessel wall also has to withstand blood pressure, which results in tension in the vessel wall. Wall stress and shear stress exert important physiological effects on the vascular cells through the process of mechanotransduction, resulting in both acute and chronic adaptations of the vascular caliber. For example, the ECs continuously sense the magnitude, direction and the pulsatility of shear stress, and are able to generate vasoactive substances, such as NO, prostacyclin and H_2_O_2_ producing an acute increase in vascular diameter. The vasodilation in response to an increase in flow is termed “flow-mediated dilation,” and is well conserved across species and vascular beds, although the magnitude of endothelium-dependent dilation and the underlying mechanism depends strongly on the species, vascular bed, vessel size, and age (Beyer et al., 2017). Arterial shear stresses range from 10 dyn/cm^2^ in the aorta to 50 dyn/cm^2^ in smaller arterioles, while the venous system has lower shears from 1 dyn/cm^2^ in the vena cava to approximately 20 dyn/cm^2^ in the venules (Papaioannou and Stefanadis, 2005; Givens and Tzima, 2016). Additionally, *in vivo*, depending on the vessel geometry (size, curvature, presence of a bifurcation or a coronary obstruction), the blood flow exhibits different patterns, falling into two major categories; laminar and disturbed flow. Laminar flow, characterized by mainly unidirectional uniform flow, occurs mostly in straight vascular segments, inducing EC alignment in the direction of flow with low cellular turnover. Pulsatile or steady laminar flow was shown to stimulate the production of factors supporting endothelial survival, quiescence and barrier function and increases the expression of anti-inflammatory genes with atheroprotective properties (Chatzizisis et al., 2007; Reinhart-King et al., 2008). Disturbed flow, also termed atheroprone flow, is characterized by oscillatory, turbulent and low flow patterns, present in vascular areas with bifurcations and curvatures (Cheng et al., 2006). ECs subjected to disturbed flow do not align in the flow direction and show increased proliferation and proinflammatory gene profiles (Chatzizisis et al., 2007) resulting in increased endothelial permeability and enhanced monocyte adhesion (Zhou et al., 2014). The mechanisms involved in flow-mediated dilation are also dependent on size; conduit vessels rely primarily on NO while the microcirculation utilizes a variety of mediators, including NO, prostacyclin and EDH. Additionally, the complex arterial architecture, with branching points and curved regions, induces different flow and shear patterns on the endothelium, greatly influencing EC function (Davies, 2009).

Additionally, the effects of cyclic stretch and pressure gradient across the endothelial layer (transmural pressure) also induce endothelial deformation, formation of endothelial ridges and alterations in endothelial function, as well as reorientation of VSMC contractile filaments from the circumferential to radial direction (Greensmith and Duling, 1984). These architectural changes may influence the pressure-induced myogenic activation and VSMC constriction leading to acute or chronic alterations in vascular responses (Lockette et al., 1986; Luscher et al., 1987). The myogenic response is the main mechanism allowing the coronary circulation to maintain constant blood flow in the face of changes in perfusion pressure, i.e., coronary autoregulation. This is achieved by constriction of coronary resistance vessels in response to an increase in intravascular distending pressure and dilation in response to a decrease in pressure. Arterioles of ~100 μm in diameter are particularly sensitive to developing myogenic tone. This response is endothelium-independent and involves VSMC activation by changes in the intracellular Ca^2+^ concentration (Kuo et al., 1988; Sorop et al., 2003), mainly via L-type Ca^2+^ channels (Jensen et al., 2017). Additionally, regional differences in myogenic tone have been found across the ventricular wall. Thus, due to the lower perfusion pressure (as a result of the resistance of the transmurally penetrating vessels and the increased extravascular compression), the sub-endocardial resistance arterioles show reduced myogenic responses as compared to subepicardial arterioles (Kuo et al., 1988). Mechanosensing of both flow and pressure is thought to result from multiple cellular components as will be discussed later.

### Regulation of Vascular Structure and Architecture

Vasodilator reserve of the microvasculature is computed as the ratio between maximal hyperemic flow and basal flow. The maximal flow is strongly dependent on the vascular architecture, the biomechanical characteristics of the vascular segments and their number (arterial and capillary density). Biomechanical properties of the vasculature play an important role in determining the minimal and maximal vascular resistance by limiting the vasoconstrictor and vasodilator reserve of the coronary vasculature. The vasoactive responses are limited by the thickness and structure of the vascular layers, in particular the organization of the VSMC layers and the extracellular matrix. The vascular structure is controlled by local forces and it is thought that shear stress, induced by the local flow profile and the wall stress related to the blood pressure, play a major role (Jones et al., 1993a,b). Additionally, chronic alterations in vascular tone induced by vasoactive substances can also result in changes in the vascular wall structure.

**Figure 2:**
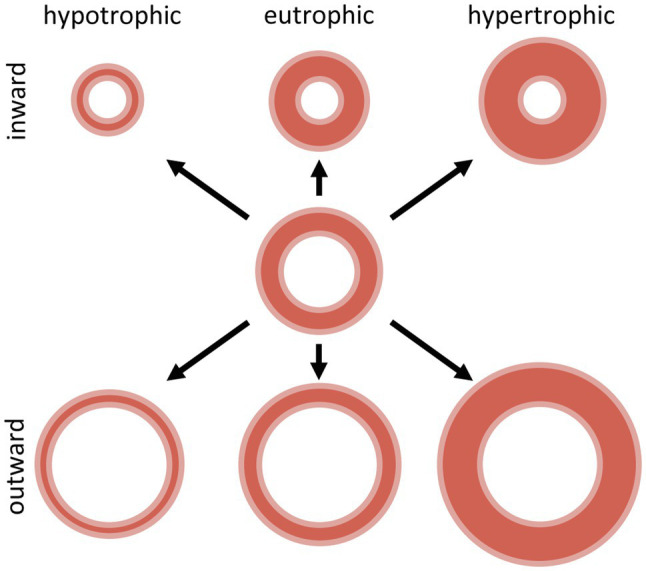
Schematic representation of inward and outward hypotrophic, eutrophic and hypertrophic vascular remodeling. Adapted with permission from Mulvany et al. (1996).

Alterations in the vascular structure are termed “remodeling” of the vessel wall and have been classified (Mulvany et al., 1996) in either eutrophic, hypotrophic or hypertrophic remodeling, depending on the changes in the wall cross-sectional area (Figure 2). Eutrophic remodeling describes vascular remodeling with preserved wall cross-sectional area around a smaller or larger lumen, hypotrophic remodeling involves loss of vascular wall components, while in hypertrophic remodeling the cross-sectional area of the vascular wall increases. Furthermore, depending on the changes in lumen diameter, vascular remodeling can be categorized as inward or outward remodeling, where a decrease in vascular lumen denotes inward remodeling while the opposite is called outward remodeling (Figure 2). Vascular remodeling is thus related to the amount of tissue in the vascular wall, and its organization around the luminal diameter, affecting the biomechanical properties and limiting the distensibility and thus the luminal diameter of that specific vascular segment. However, there is evidence that even under normal circumstances, the vessel wall is not quiescent, as vessels undergo continuous turnover of wall components, thereby contributing to homeostasis (Van Den Akker et al., 2010).

Most studies assessing small artery remodeling have been performed *in vitro* in isolated vessels or based on histological examination (measurement of cross-sectional areas) of vascular segments fixated under pressure (Bakker et al., 2004, 2005; Van Den Akker et al., 2010; Tuna et al., 2013). While the latter approach might seem more physiologically relevant, since the vessels are not removed from their surrounding tissue, a direct comparison with the “pre-diseased” state is difficult and one-to-one comparison to similar healthy vessels from the exact same location is impossible. The *in vitro* approach (based on diameter lumen measurements in conjunction with optical/histological assessment of the wall/lumen ratio) has been used by many research groups, and enables the study of the vascular segment prior to and following exposure to the mechanical stimulus needed to induce the remodeling response. Passive pressure-diameter curves performed at different points in time allow for the observation of the process dynamics and a more complete characterization of the remodeling type. Additionally, the *in vitro* set-up is ideal to study the mechanisms underlying vascular remodeling. Conversely, removal of the vessel from the myocardium completely abolishes the natural contribution of any metabolic stimuli to this process.

Using *in vitro* culture of isolated small arteries, it has been shown that chronic vasoconstriction was sufficient to induce vascular inward remodeling, while vasodilation did not affect the vascular diameter or even resulted in outward remodeling (Bakker et al., 2002, 2003; Pistea et al., 2005, 2008; Sorop et al., 2006). Additionally, even in the absence of active constriction, vessels cultured at low pressures maintaining a small lumen diameter remodeled in the course of a couple of days, whereas pharmacological vasodilation significantly attenuated the inward remodeling (Sorop et al., 2006). Importantly, vessels cultured under flow remained more dilated than vessels cultured without flow, and flow inhibited the inward remodeling (Pistea et al., 2005). Bakker et al. studied the mechanisms involved in wall stress-induced vascular remodeling in more detail (Bakker et al., 2005), demonstrating tissue transglutaminase (TG2) as one of the crucial mediators of crosslinking between the extracellular matrix proteins, limiting vascular distensibility. Several factors have subsequently been shown to be involved in modulating TG2 activity, including NO, Ca^2+^ and GTP/GDP concentrations, but also the redox state in the micro-environment (Van Den Akker et al., 2010; Del Campo et al., 2013; Huelsz-Prince et al., 2013). Bakker et al., (2005) postulated that the mechanical force exerted by VSMCs during contraction, directly activates TG2, which further results in crosslinking of extracellular matrix components, explaining the link between smooth muscle activation and inward remodeling (Bakker et al., 2005). Additionally, *in vitro* studies also indicate that matrix metalloproteinases (MMPs) are activated in VSMCs subjected to either stationary stretch (Meng et al., 1999; Asanuma et al., 2003; Lehoux et al., 2004) or cyclic stretch (Grote et al., 2003), or in arteries exposed to longitudinal tension (Jackson et al., 2002), possibly contributing to a continuous turnover of matrix elements and counterbalancing the activation of TG2. Distal to small arteries and arterioles, and strongly dependent on the activation and remodeling status of these proximal vessels, the capillary network is involved in the oxygen delivery to the cardiomyocytes. Although its contribution to the total vascular resistance is below 10%, (Chilian et al., 1986), alterations in capillary density, especially in the subendocardium, have important consequences for local myocardial function. In order to compensate for a higher oxygen demand and lower perfusion pressures, the subendocardial capillary to fiber ratio is higher (~1) than in the subepicardial layer, (~0.8); however, these ratios have been shown to be strongly affected by different pathologies (Duncker and Bache, 2008). Loss of microvascular density, i.e., vascular rarefaction, is a key component of various pathologies. Factors contributing to capillary rarefaction include removal of angiogenic stimuli or generation of anti-angiogenic substances, flow discontinuation, disruption of endothelial-pericyte association and endothelial dysfunction (Goligorsky, 2010). It is important to realize that different disease entities, particularly metabolic syndrome, hypertension and aging, as well as the presence of a proximal obstruction, resulting in metabolic and/or hemodynamic alterations, can have a large impact on both function and structure of the coronary microvasculature, and hence on vascular resistance and myocardial perfusion. The impact of these diseases on the microvascular structure and architecture will be discussed below.

### Biomechanical Signaling in Microvascular Function and Remodeling

**Figure 3:**
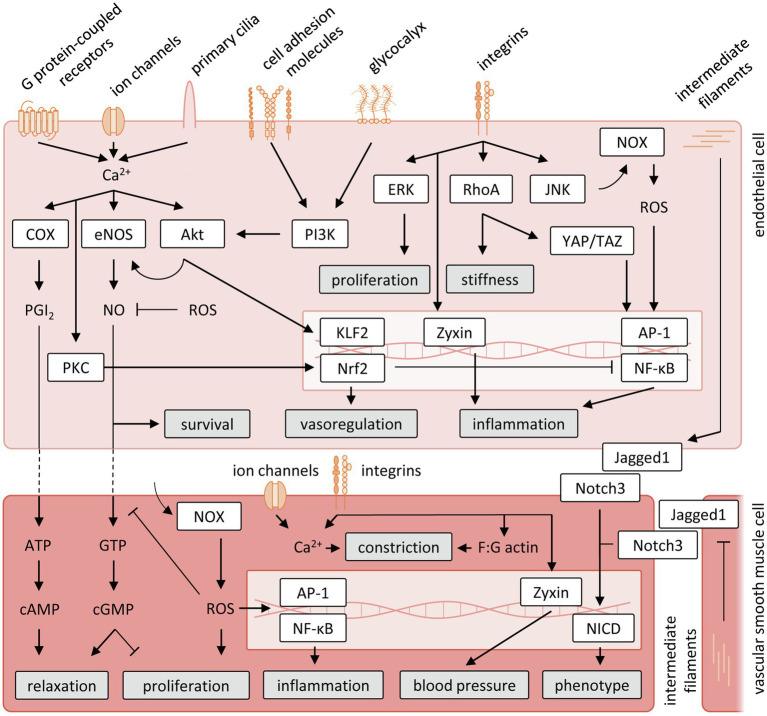
Schematic representation of vascular mechanosensing and signal transduction cascades. Akt, protein kinase B; AP-1, activator protein 1; ATP, adenosine triphosphate; cAMP, cyclic adenosine monophosphate; cGMP, cyclic guanosine monophosphate; COX, cyclooxygenase; eNOS, endothelial nitric oxide; ERK, extracellular signal-regulated kinases; GTP, guanosine triphosphate; JNK, jun N-terminal kinase; KLF2, krüppel-like Factor 2; NF-κB, nuclear factor-κB; NOX, nicotinamide adenine dinucleotide phosphate oxidase; NO, nitric oxide; Nrf2, nuclear factor erythroid 2-like 2; PGI2, prostacyclin; PI3K, phosphatidylinositol 3-kinase; PKC, protein kinase C; RhoA, Ras homolog family member A; ROS, reactive oxygen species; YAP/TAZ, yes-associated protein/transcriptional coactivator with PDZ-binding motif.

Chronic increases or decreases in shear stress and wall tension have been shown to induce vessel remodeling to maintain proper tissue perfusion. Changes in shear stress lead to proportional changes in vascular diameter to the extent that the original levels of shear stress are restored (Langille, 1996; Tuttle et al., 2001; Baeyens and Schwartz, 2016), suggesting shear stress to be a set variable controlled by endothelial-mediated dilation and remodeling. Given the important influence of mechanical forces on vascular function and structure, the understanding of mechanosensing mechanisms by which ECs and VSMCs convert physical stimuli to biological responses has been an active field of research, although data exclusively for the coronary circulation are limited and to date these mechanisms have been studied mainly *in vitro*. Below, we will discuss data obtained in different vascular beds, on the main molecular contributors to mechanosensing. The pathways discussed are summarized in Figure 3.

#### Ion Channels

Ion channels are pore-forming proteins regulating the distribution of ions across the cell membrane, thereby enabling the establishment of a resting membrane potential. A large variety of ion channels is expressed by coronary microvascular ECs (Kefaloyianni and Coetzee, 2011) and VSMCs (Tykocki et al., 2017). For many of these ion channels, it has been shown that both shear stress and cyclic stretch can trigger their reversible deformation, directly affecting the opening of these channels and thereby the translation of mechanical stimuli into biomechanical signaling (Folgering et al., 2008). Well described ion channels sensing vascular mechanical stimuli include transient receptor potential (TRP) channels, Piezo channels and the epithelial sodium channel (ENaC).

The TRP family consists of conserved membrane proteins that mostly function as non-selective cation channels. Many of these TRP channels, including TRPC1, TRPC6, TRPV4, TRPM7 and TRPP2, are involved in mechanical stress-induced signaling by raising intracellular Ca^2+^ levels in ECs (Inoue et al., 2009), resulting in activation of calmodulin and subsequent production of prostacyclin (PGI_2_) and NO. In VSMCs, PGI_2_ induces relaxation via protein kinase A (PKA) after conversion of adenosine triphosphate (ATP) into cyclic adenosine monophosphate (cAMP). Similarly, NO stimulates the conversion of guanosine triphosphate (GTP) in VSMCs into cyclic guanosine monophosphate (cGMP), leading to protein kinase G (PKG) activation and VSMC relaxation (Kohler and Hoyer, 2007). In addition, in some—but not all—vascular beds there is also evidence for spreading of the endothelial hyperpolarization into neighboring VSMCs via myoendothelial gap junctions, closing L-type voltage-gated Ca^2+^ channels leading to vasorelaxation (Sandow et al., 2002; Conejo et al., 2007). Conversely, wall stress-induced Ca^2+^ influx via TRP channels in VSMCs results in membrane depolarization, stimulating vasoconstriction (Hill-Eubanks et al., 2014).

In addition to TRP channels, Piezo channels have also frequently been linked to mechanosensing and subsequent signaling. Using short interference RNA-mediated knockdown studies in a neuroblastoma cell line, Piezo1 and Piezo2 were shown to act as mechanically activated cation channels (Coste et al., 2010). In later studies, overexpression of Piezo1 in non-mechanically responsive human embryonic kidney (HEK) 293T cells was demonstrated to result in elevated Ca^2+^ influx upon exposure to shear stress (Dubin et al., 2017). Conversely, knockdown of Piezo1 in ECs largely prevented shear-induced endothelial alignment, indicating that Piezo1 plays a prominent role in the structural remodeling of the vasculature in response to mechanical stimuli (Ranade et al., 2014). Piezo1 was shown to initiate a rapid influx of Ca^2+^ in ECs when exposed to shear, similar to TRP channels inducing vasodilation via NO (Wang et al., 2016a; Jin et al., 2021). Besides stimulation of endothelial nitric oxide synthase (eNOS) activity, Piezo1 and TRP channels are also involved in shear-induced transcriptional regulation. Loss of TRPV4 or Piezo1 results in reduced activation of krüppel-like factor 2 (KLF2; Gerhold and Schwartz, 2016). KLF2, in concert with nuclear factor erythroid 2-like (Nrf2), regulates shear-induced transcription of many genes involved in vasoregulation (enhanced transcription of eNOS and C-natriuretic peptide, reduced ET-1 and angiotensin-converting enzyme transcription), inflammation [reduced transcription of inflammatory genes by blocking the activity of nuclear factor-κB (NF-κB) and activator protein 1 (AP-1)] and oxidative stress (enhanced transcription of antioxidant genes such as NAD(P)H dehydrogenase quinone 1 and catalase), and as such, acts as an important determinant of vascular remodeling (Novodvorsky and Chico, 2014).

Epithelial sodium channel is a sodium selective ion channel that has primarily been studied in cells of the distal nephrons in the kidney (Bhalla and Hallows, 2008). More recently, endothelial influx of sodium ions via ENaC was shown to regulate cellular actin dynamics. Enhanced activation, which occurs in response to acute changes in shear stress, results in stabilization of cortical actin, a thin actin mesh directly underneath the plasma membrane, into its filamentous form (F-actin), producing a stiffer cell-cortex (Warnock et al., 2014).

#### Cytoskeleton and Intermediate Filaments

Intermediate filaments, together with actin filaments and microtubules, comprise the cytoskeleton. Critical to a multitude of cell functions, including the maintenance of cell shape and organization, as well as facilitation of cell migration and protein and vesicle trafficking, the cytoskeleton is also recognized as an important mechanosensing and mechanotransduction structure.

As part of the cytoskeleton, the intermediate filament Vimentin has unique strain stiffening behavior, with Vimentin filaments being flexible at low stretch, but become more rigid at high stretch levels (Antfolk et al., 2017). This contributes to stabilization of the intracellular environment when cells are exposed to high mechanical forces, thereby stimulating cell survival and function. Additionally, in mesenteric resistance arteries, Vimentin was shown to be required for flow-mediated vasodilation (Henrion et al., 1997). In isolated ECs, Vimentin responded to shear stress by increased phosphorylation at serine 38, resulting in stabilization of Jagged1 on the endothelial membrane and subsequent Notch3 binding and signaling in VMSCs. In VSMCs, exposure to cyclic stretch promotes Vimentin polymerization, reduced Jagged1 expression and subsequent reduced Notch3 signaling in neighboring VSMCs (Van Engeland et al., 2019). Binding of Jagged1 to Notch3 receptor triggers cleavage and release of the Notch intracellular domain (NICD), which translocates into the nucleus to act as a transcription factor for target genes, including Hes1 and Hey1, that regulate vascular cell behavior. During development, Notch activation by ECs guides mural cell recruitment to sprouting vessels and VSMC differentiation (Stassen et al., 2020). Vimentin was shown to regulate Notch signaling during angiogenesis by consolidating the Jagged1/Notch signaling response at the expense of Delta-like ligand 4 (DLL4)/ Notch signaling (Antfolk et al., 2017). In mature arteries, Notch signaling between ECs and VSMCs, as well as between different layers of VSMCs, plays a central role in guiding the adaptation of the media in response to mechanical stimuli, regulating VSMC contractile versus synthetic phenotype, as well as proliferation and survival (Loerakker et al., 2018; Morris et al., 2019; Stassen et al., 2020).

#### Cell Adhesion Molecules

Cell adhesion molecules (CAMs) play an important role in maintaining tissue structure by providing physical cell-to-cell and cell-to-matrix adherence. Several prominent endothelial CAMs, including platelet endothelial cell adhesion molecule-1 (PECAM-1) and vascular endothelial cadherin (VE-cadherin), both constitutively expressed in virtually all ECs, were shown to be key players in mechanotransduction. PECAM-1 is a transmembrane receptor belonging to the immunoglobulin superfamily which provides endothelial cell-to-cell adhesion via homophilic interaction with surrounding ECs (Paddock et al., 2016). Application of both shear and stretch has been shown to produce tension on PECAM-1 resulting in rapid PECAM-1 phosphorylation (Fleming et al., 2005), which was independent of shear- and stretch-induced Ca^2+^ influx but rather involves tyrosine-protein kinase Fyn resulting in recruitment of both growth factor receptor-bound protein 2 (GRB2)-associated binding protein 1 and Src homology region 2 domain-containing phosphatase-2 (SHP-2). Upon phosphorylation of PECAM-1, the recruited SHP-2 initiates rapid but temporal extracellular signal regulated kinase (ERK) 1/2 activation, regulating cell proliferation (Xu et al., 2016). Similar to PECAM-1, VE-cadherin is also a transmembrane glycoprotein providing cell-to-cell adherence via homophilic interactions (Brasch et al., 2011). Using antibody-coated magnetic beads, force application on VE-cadherin resulted in cellular stiffening and cytoskeletal reorganization, illustrating the mechanical stress sensing abilities of VE-cadherin (Barry et al., 2015). However, exposing a mixture of wildtype and VE-cadherin knockout ECs to flow, prompted alignment (in the direction of flow) of VE-cadherin expressing cells, even in the absence of homophilic adhesion, which suggests that VE-cadherin acts as an adaptor, rather than as receptor, in this response (Tzima et al., 2005). This was confirmed in non-vascular COS-7 cells, which only aligned to flow when co-transfected with plasmids encoding PECAM-1, VE-cadherin and vascular endothelial growth factor receptor 2 (VEGFR2; Tzima et al., 2005). Other studies indicated that, in response to flow, VE-cadherin links PECAM-1 to VEGFR2 leading to ligand-independent activation of VEGFR2 and subsequent activation of phosphatidylinositol-3-OH kinase (PI3K; Jin et al., 2003). PI3K further activates serine/threonine kinase protein kinase B (Akt) which stimulates eNOS-induced production of vasodilatory NO, as well as ERK5-mediated dissociation of histone deacetylase 5 (HDAC5) from myocyte enhancer-binding factor 2 (MEF2), leading to enhanced shear-induced transcription of KLF2 (Chistiakov et al., 2017). Additionally, PI3K inhibits binding and subsequent degradation of Nrf2 by kelch-like ECH-associated protein 1 (KEAP-1; Dai et al., 2007).

Integrins, another class of CAMs, are transmembrane receptors that facilitate the connection between the extracellular matrix and the cytoskeleton, like intermediate filaments, putting them in a unique position to transmit physical stimuli. They are heterogenous in structure, being composed of various combinations of α and β subunits. Direct assessment of endothelial integrin conformational changes, as well as antibody-mediated blockade of the shear-induced response, has provided evidence for the involvement of integrins in mechanical signal transduction. Shear stress-induced changes in integrin conformation increase their affinity for extracellular matrix proteins, such as fibronectin, laminin and collagen (Shyy and Chien, 2002), binding to these substrates leading to the formation of focal adhesion complexes, which link the actin cytoskeleton to the extracellular environment and simultaneously stimulate the activation of focal adhesion kinase (FAK; Fang et al., 2019). Via activation of Rap1 guanine exchange factor C3G, FAK stimulates the activation of ERK, thereby enhancing cellular proliferation and migration (Shyy and Chien, 2002). Furthermore, in isolated ECs and VSMCs it has been demonstrated that in response to cyclic stretch, focal adhesion protein Zyxin dissociates from the focal adhesion complex and accumulates in the nucleus (Cattaruzza et al., 2004; Wojtowicz et al., 2010), enhancing transcription of inflammatory genes in ECs and upregulating the ET-1 B receptor (ET_B_-R) in VSMCs. Elevation of intravascular pressure also results in enhanced filamentous:globular (F:G) actin ratios in VSMCs. This polymerization of actin aids in the development of myogenic tone, as evidenced by impaired constriction of VSMCs exposed to actin polymerization inhibitors cytochalasins and latrunculin. The exact mechanism by which strain induces actin polymerization in VSMCs is not completely understood, but it most likely occurs via integrin-mediated activation of protein kinase C (PKC) and RhoA (Cipolla et al., 2002).

Additionally, activation of endothelial integrins also occurs indirectly upon exposure to shear. PI3K activation by the tri-molecular complex composed of PECAM-1, VEGFR2 and VE-cadherin leads to an integrin-mediated, substrate-dependent mechanotransduction response. Thus, PI3K-mediated activation of integrins bound to collagen initiate a protein kinase A-dependent repression of RhoA, leading to reduced synthesis of cellular stress fibers and endothelial stiffness. Conversely, activation of integrins bound to fibronectin stimulates RhoA-mediated stiffness and enhances activation of inflammatory signaling via NF-κB (Collins et al., 2014). Moreover, integrin-mediated activation of RhoA also stimulates yes-associated protein (YAP) and transcriptional coactivator with PDZ-binding motif (TAZ), initiating the transcription of a variety of inflammatory genes by activating AP-1 via jun n-terminal kinase (JNK; Wang et al., 2016b).

#### G Proteins and G Protein-Coupled Receptors

G proteins are membrane-bound guanine nucleotide-binding proteins acting as molecular switches to enable the transmission of external stimuli into the cell. They can be subdivided in monomeric small GTPases and heterotrimeric G protein complexes consisting of an α, β and γ subunit, both of which are regulated in their activity by their ability to bind and hydrolyze guanosine triphosphate (GTP) to guanosine diphosphate (GDP). Heterotrimeric G proteins have previously been shown to operate as mechanosensitive initiators of signaling. Onset of flow rapidly induced GTP binding in human umbilical vein ECs (HUVECs; Gudi et al., 1996). Using G protein-loaded phospholipid bilayer vesicles, it was later demonstrated that shear-induced activation of G proteins (G_αq_ and G_αi3_) was independent of an intact cytoskeleton or receptor-mediated signaling (Gudi et al., 1998). Short interference RNA-mediated knockdown of G_αq_ illustrated its requirement in shear-induced activation of endothelial Ras, implicating a direct role for G protein signaling in mitogen-activated protein kinase (MAPK) activity (Gudi et al., 2003).

There are, however, also studies showing evidence for biomechanical G protein-coupled receptor signaling (GPCR). It has, for instance, been observed that application of shear, stretch or a membrane-fluidizing agent to bovine aortic ECs, resulted in a rapid and ligand-independent conformation change of the bradykinin B_2_ GPCR (Chachisvilis et al., 2006), known to activate MAPKs and to initiate phospholipase-mediated increase in intracellular Ca^2+^, leading to eNOS activation. Similarly, activation of GPCR sphingosine-1 phosphate (S1P) receptor-1, which like bradykinin B_2_ GPCR is expressed by coronary ECs (Figueroa et al., 2001; Liu et al., 2016), was shown to induce flow-dependent activation of ERK, Akt and eNOS. In line with activation of bradykinin B_2_ GPCR, activation of S1P receptor-1 is ligand-independent as ligand binding-deficient mutants of S1P receptor-1 were able to functionally restore the shear-mediated actions of S1P receptor-1 knockout in HUVECs (Jung et al., 2012).

More recently, G Protein-Coupled Receptor 68 (GPR68), which is primarily expressed in ECs of small diameter arteries (murine third-order mesenteric), has also been suggested to be a flow responsive GPCR, as indicated by the shear-induced Ca^2+^ influx in HEK293T cells overexpressing GPR68. *Ex vivo* cannulation experiments comparing the shear response of third-order mesenteric arteries from wildtype and GPR68 knockout mice, demonstrated an impaired flow-mediated dilation in absence of GPR68. Moreover, eNOS inhibition could almost completely block flow-mediated dilation in arteries of both wildtype and knockout mice, indicating that GPR68 presumably functions upstream of the NO pathway (Xu et al., 2018).

Interestingly, GPCR-mediated signaling upon mechanical stress has also been described in VSMCs. Exposure of VSMCs to hypo-osmotic shock to increase membrane tension, resembling mechanical stretch, was shown to result in increased intracellular Ca^2+^ levels causing VSMC contraction. Remarkably, this influx of Ca^2+^ could be blocked by losartan, an inhibitor of the GPCR angiotensin II receptor type 1 (AT1-R; Schleifenbaum et al., 2014).

#### Glycocalyx

The glycocalyx is a thin (up to 500 nm), negatively charged, gel-like structure on the luminal side of the membrane of healthy endothelium, and as such, it has also been observed in the coronary circulation (Becker et al., 2010). It is composed of proteoglycans and glycoproteins, such as heparan sulfate and hyaluronic acid. It regulates endothelial barrier function, leukocyte adhesion and coagulation, but has also been linked directly to mechanotransduction. Thus, Syndecan-1 and -4, heparin sulfate and proteoglycans, which attach the glycocalyx to the cytoskeleton, were shown to be indispensable for proper shear-induced activation of Akt. Moreover, loss of endothelial syndecan expression impaired endothelial alignment to the direction of flow, lowered the transcription of flow-induced transcription factors KLF2 and KLF4, and stimulated the transcription of a variety of pro-inflammatory cytokines (Voyvodic et al., 2014). The glycocalyx also plays a role in flow-mediated dilation, as demonstrated by heparin challenge-induced displacement of proteins bound to heparan sulfate proteoglycans in mice, which led to impaired arteriolar vasodilation during reactive hyperemia (Vanteeffelen et al., 2007). Similarly, *ex vivo* analysis of rat mesenteric arteries loaded with a fluorescent NO indicator illustrated that enzymatic removal of heparan sulfate abolished flow-induced NO synthesis (Yen et al., 2015). Moreover, application of mechanical stretch to microvascular ECs was shown to induce Ca^2+^-dependent NO production, but only in the presence of heparan sulfate and hyaluronic acid (Dragovich et al., 2016).

There is also evidence linking the actions of individual components of the glycocalyx to PECAM-1-mediated mechanosignaling. It has been shown that PECAM-1 associates with G protein G_aq/11_ in ECs exposed to laminar shear and that pharmacological inhibition, as well as enzymatic removal of heparan sulfate abrogated this association (Dela Paz et al., 2014). Although the exact consequence of the association between these proteins is not completely understood, it illustrates how mechanosensors apparently act in parallel, perhaps even interact, rather than operate individually.

#### Primary Cilia

Primary (non-motile) cilia are hair-like protrusions of the apical cell membrane, structurally composed of 9 microtubule doublets that are directly linked to the intracellular cytoskeleton. Most mammalian cell types, including coronary ECs (Singh et al., 2020), possess these non-motile cilia. However, the length of these cilia is variable, with ECs exposed to high shear stress tending to have shorter cilia than ECs exposed to relatively low shear stress (Mohieldin et al., 2016). Evidence for the involvement of cilia in mechanosignaling came initially from a study subjecting ECs isolated from *Tg737* mutant mice, an orthologous gene of intraflagellar transport 88 (IFT88, involved in cilium biogenesis), to flow (Nauli et al., 2008). Thus, *Tg737* mutant ECs, lacking functional cilia, were unable to initiate shear-induced NO synthesis. Further studies indicated that a complex of polycystin-1 (PC1), a transmembrane glycoprotein regulating the function of the Ca^2+^ permeable cation channel polycystin-2 (PC2), is responsible for the intracellular conversion of the flow-mediated activation of cilia. Both PC1 and PC2 are particularly enriched in the ciliary membrane and knockout of either PC1 or PC2 is sufficient to block shear-mediated Ca^2+^ influx and subsequent NO production by eNOS (Aboualaiwi et al., 2009). The polycystin-mediated influx of Ca^2+^, in addition to shear-induced intracellular Ca^2+^ release via other mechanotransduction routes, also activates PKC (Saternos and AbouAlaiwi, 2015). PKC, in turn, phosphorylates Nrf2 at Ser-40, which—in concert with the PI3K-mediated dissociation of Nrf2 from KEAP-1—stimulates shear-induced transcriptional activation of Nrf2 (Huang et al., 2002; Hsieh et al., 2009).

## Coronary Microvascular Function and Structure in Disease

In more than 50% of patients with coronary artery disease, percutaneous coronary intervention re-establishes coronary artery patency, however without completely restoring myocardial perfusion (Uren et al., 1993a,b; Niccoli et al., 2009, 2010) indicating that microvascular dysfunction is a critical contributor to ischemia distal to an epicardial stenosis. Risk factors, such as metabolic dysregulation and diabetes, age, and hypertension present in a large proportion of the patients with coronary artery disease and have been shown to impact both the macro- and microvasculature (Padro et al., 2020; Sorop et al., 2020). Among the factors involved, endothelial dysfunction—significantly impacted by these risk factors—appears to be an important contributor to the development of micro- and macrovascular disease (Sorop et al., 2020; Van De Wouw et al., 2020, 2021). In addition, changes in hemodynamic factors inducing vascular remodeling (Sorop et al., 2008; Weil et al., 2020), as well as microvascular rarefaction (Sorop et al., 2018; Van De Wouw et al., 2020) may also play a role in the co-existence of macro- and microvascular disease. While microvascular endothelial dysfunction has been studied more extensively in this context, the mechanical determinants of the microvascular architecture and their interaction are less well understood.

**Figure 4:**
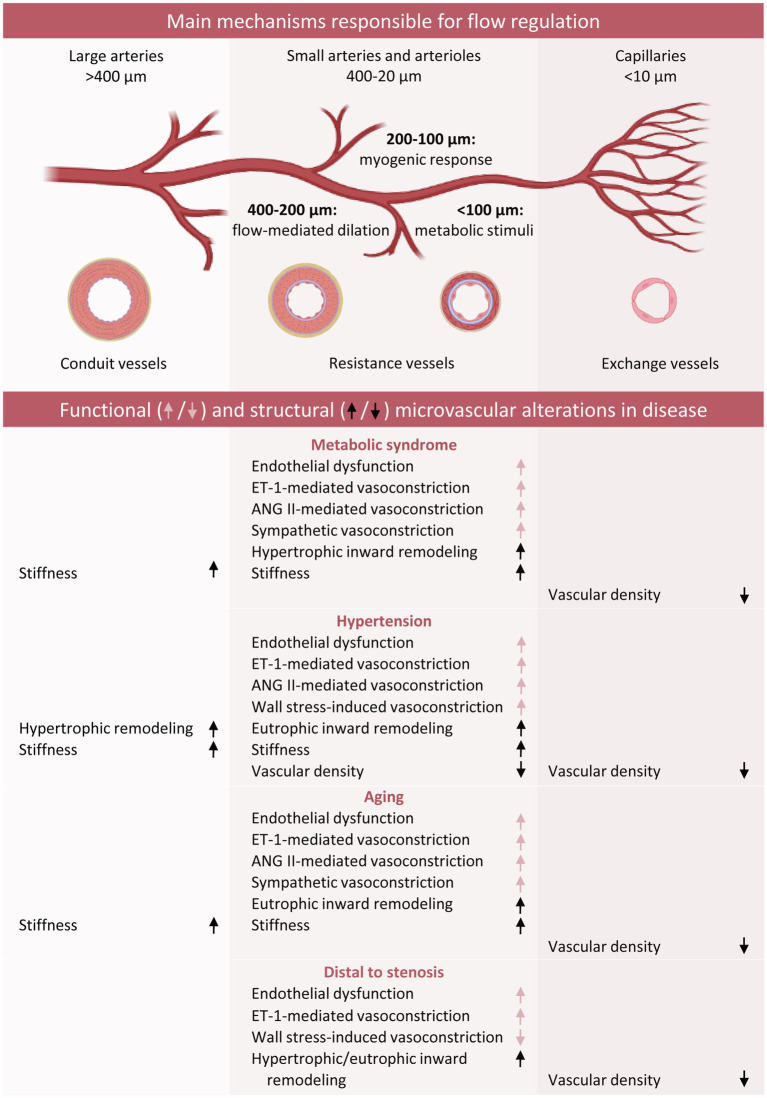
Schematic overview of functional and structural coronary microvascular alterations in the presence of classic risk factors. Red arrows refer to changes in vascular function, whereas black arrows refer to vascular remodeling. ANG II, Angiotensin II; ET-1, endothelin-1.

Indeed, the presence of a proximal coronary obstruction, resulting in alterations in distal pressure and flow (Hoogendoorn et al., 2020; De Nisco et al., 2021), has also been shown to impact the distal microvasculature both at the functional and structural level, and the presence of risk factors may exacerbate these effects. On the other hand, episodes of myocardial ischemia may trigger angiogenesis from adjacent regions with still intact perfusion as well as outward remodeling of pre-existing connecting vessels (arteriogenesis) in a process of collateralization. This allows for a, at least partial, restoration of myocardial blood flow to the area distal to the stenosis. We will discuss below how different pathologies, such as the presence of cardiovascular risk factors and a chronic coronary artery stenosis contribute to development and/or aggravation of microvascular dysfunction and microvascular remodeling including diameter and density alterations (Figure 4). Furthermore, our current understanding of the mechanisms involved in these processes initiated by, or interfering with, biomechanical signaling will be addressed.

### Metabolic Dysregulation

Metabolic dysregulation includes conditions such as metabolic syndrome, obesity, insulin resistance, diabetes mellitus, hypercholesterolemia and hypertriglyceridemia, that either alone or in combination contribute to coronary artery disease and stroke (Cho et al., 2018).

Clinical and experimental studies have shown that metabolic dysregulation is associated with perturbations in CBF control during increased metabolic demand (Berwick et al., 2012; Paneni et al., 2013; Crea et al., 2014; Duncker et al., 2015; Badimon et al., 2017; Van De Wouw et al., 2020). Indeed, metabolic syndrome and obesity are associated with impaired coronary flow reserve (CFR; Di Carli et al., 2003; Pirat et al., 2008; Zorach et al., 2018), which also worsens with the onset of type 2 diabetes (Kondo et al., 2001; Schindler et al., 2006). These studies are supported by observations in dogs (Setty et al., 2003) and swine with co-morbidities (Bender et al., 2016; Van De Wouw et al., 2020), demonstrating progressive impairment of myocardial oxygen delivery during graded treadmill exercise, suggesting that the mechanisms responsible include both microvascular dysfunction and remodeling. Indeed, acute hyperglycemia in young subjects impaired adenosine-mediated increase in CBF (Di Carli et al., 2003). Additionally, swine subjected to 2.5 months of hyperglycemia and hypercholesterolemia showed impaired endothelial function of isolated coronary small arteries, mediated via loss of NO, despite preserved VSMC function (Van Den Heuvel et al., 2012). Indeed, hyperglycemia and hypercholesterolemia can induce a state of oxidative stress, which is a state in which the formation of reactive oxygen species (ROS), including superoxide anion, hydroxyl anion or H_2_O_2_ exceeds the antioxidant defense mechanisms. Superoxide anions can directly interact with NO, thereby limiting NO bioavailability and resulting in the formation of the pro-inflammatory peroxynitrite. In the same animal model studied 15 months after induction of hyperglycemia and hypercholesterolemia (Sorop et al., 2016), increased vasoconstrictor response to ET-1 was observed, which was ET_B_-mediated. Surprisingly, the endothelium-dependent vasodilation to bradykinin was no longer reduced as compared to control, although the contribution of EDH to the bradykinin-induced dilation was reduced (Sorop et al., 2016). Interestingly, at this stage, these microvascular alterations were also observed in non-diabetic, hypercholesterolemic swine. Similarly, obesity has also been shown to increase coronary microvascular sensitivity to vasoconstrictors (ET-1, prostaglandin H2 or thromboxane A_2_) in animal models (Berwick et al., 2012) and humans (Barton et al., 2012; Campia et al., 2012). In addition to endothelial dysfunction, VSMC function is also affected by metabolic dysregulation, as in obese Ossabaw swine with metabolic syndrome, 16 weeks of high fat diet resulted in increased coronary vasoconstriction mediated by altered electromechanical coupling between K_V_ and Ca_V1.2_ channels in VSMCs (Berwick et al., 2013).

A metabolic dysregulation-associated increase in sympathetic activity has also been documented both in patients and animal models, resulting in exaggerated alpha-adrenergic coronary vasoconstriction (Huggett et al., 2004; Dincer et al., 2006). Additionally, substantial evidence indicates that activation of the renin angiotensin aldosterone system (RAAS) associated with adipose tissue-derived angiotensinogen, as well as adipocyte-derived free fatty acids (FFAs) and leptin, results in microvascular dysfunction and impaired CFR, possibly due to increased angiotensin II-mediated vasoconstriction (Kachur et al., 2018). Indeed, perivascular adipose tissue-derived adipokines such as leptin, resistin, IL-6 and TNF-α are potent pro-inflammatory molecules promoting oxidative stress in the endothelium and altering endothelial function and NO bioavailability, either directly or via increased ET-1 production.

Furthermore, leptin derived from perivascular fat also promotes coronary arterial vasoconstriction and VSMC proliferation via Rho kinase signaling (Bagi et al., 2011; Noblet et al., 2016). Additionally, adipocyte-derived circulating FFA and hyperglycemia-induced advanced glycation end products (AGE) lead to increased oxidative stress, thereby limiting NO bioavailability, as well as increased production of vasoconstrictor factors such as thromboxane A2 and ET-1 (Creager et al., 2003).

The sustained vasoconstriction induced by the various mechanisms described above may contribute to structural vascular alterations. Indeed, not only changes in microvascular function have been documented in metabolic dysregulation, but microvascular structure is also affected. In Ossabaw swine, 16 weeks of metabolic syndrome induced by a high fat/high fructose diet resulted in impaired myocardial perfusion and blunted response to adenosine, associated with reduced microvascular density (Li et al., 2012). In the same animal model, 6 months of metabolic syndrome and high-fat diet resulted in impaired hyperemic flow associated with augmented coronary myogenic tone, hypertrophic inward remodeling of the coronary resistance arteries and capillary rarefaction (Trask et al., 2012).

Such alterations can be due to the deleterious effects of the metabolic dysregulation on the molecular mechanisms involved in mechanosensing. Thus, metabolic syndrome has previously been demonstrated to be detrimental to the endothelial glycocalyx, as evidenced by shedding-induced increase of glycocalyx components in the bloodstream of type 1 and type 2 diabetic patients, thereby reducing shear-induced eNOS activity and NO production (Nieuwdorp et al., 2006; Broekhuizen et al., 2010). Recent data in obese mice also indicate that breakdown of the glycocalyx induced by metabolic derangements impairs the function of the inward rectifying K^+^ channel K_ir_2.1, thereby limiting the endothelial response to flow (Fancher et al., 2020). Additionally, metabolic syndrome-associated inflammation, FFAs and hyperglycemia have all been linked to activation of endothelial NADPH oxidase (NOX; Devallance et al., 2019), stimulating the production of ROS and uncoupling of eNOS. The consequent reduction in NO bioavailability not only affects vascular tone, but, over time, may also results in impaired cGMP-mediated inhibition of VSMC proliferation and hypertrophic remodeling (Tanner et al., 2000). Moreover, adipose tissue-derived pro-inflammatory adipokines potentiate VSMC proliferation, either directly via leptin (Oda et al., 2001), or indirectly via paracrine signaling through NF-κB-induced infiltration of macrophages (Zampetaki et al., 2005; Spescha et al., 2014). Leptin enhances the expression of collagen, fibronectin, transforming growth factor (TGF)-β and connective tissue growth factor (CTGF; Martinez-Martinez et al., 2014). In combination with decreased NO-mediated S-nitrosylation of TG2 (Jung et al., 2013), enhancing its ECM crosslinking activity, this elevated ECM transcription could contribute to the observed stiffening of small arteries in swine after 15 months of high fat diet in absence of vascular wall hypertrophy (Sorop et al., 2016).

Together with microvascular dysfunction and structural abnormalities of the vascular wall, alterations in vascular density may also contribute to the perturbations in CBF and oxygen delivery and even regional ischemia during exercise observed in humans (Camici and Crea, 2007; Bairey Merz et al., 2017) and animals with co-morbidities (Setty et al., 2003; Bender et al., 2016, van de Wouw et al., 2020). Indeed, in freshly explanted hearts of diabetic patients, lower capillary density and pericyte loss as compared to non-diabetic subjects were reported, accompanied by a lower angiopoietin 1/angiopoietin 2 ratio (Hinkel et al., 2017). Similarly, in the left ventricle of obese subjects, lower capillary densities were observed as compared to lean subjects (Campbell et al., 2013). These data were confirmed in different animal models, including obese Zucker rats (Toblli et al., 2004), obese Wistar–Kyoto rats with metabolic syndrome (Machado et al., 2017) and obese diabetic *db*/*db* mice (Gonzalez-Quesada et al., 2013). The pathways contributing to this capillary rarefaction were mostly associated with VEGF signaling, but inflammatory factors, certain miRNAs and even changes in ECM composition have also been shown to take part in metabolic derangement-induced capillary rarefaction (Paavonsalo et al., 2020).

### Hypertension

Arterial hypertension, defined as a systolic blood pressure ≥ 140 mmHg and/or a diastolic blood pressure ≥ 90 mmHg (Unger et al., 2020), continues to increase in prevalence worldwide, especially in developing countries, mainly as a result of an unhealthy lifestyle. Arterial hypertension is an independent risk factor for coronary artery disease and stroke (Chobanian et al., 2003; Benjamin et al., 2019) and frequently co-exists with other aggravating pathologies, such as diabetes and chronic kidney disease, underlying their onset and progression. An important contributor to myocardial ischemia in patients with arterial hypertension is coronary microvascular dysfunction, (Modolo et al., 2015). Indeed, antihypertensive treatment has been able to improve both basal and hyperemic flow in patients (Neglia et al., 2011). In hypertensive rats, this effect was shown to be attributable to improved microvascular function and the reverse remodeling of intramural coronary arterioles (Neglia et al., 2011).

In the microvasculature, an increased blood pressure results in elevated circumferential stress (*σ*), which, according to the law of Laplace (*σ* = *P* × *R*/*h*, where *P* is transmural pressure, *R* is vessel radius, and *h* is wall thickness), can be normalized by lowering the luminal diameter or by increasing the wall thickness. These responses, aimed to normalize wall stress, are thought to be protective in the short term. However, the increase in vascular resistance and subsequent impairment in maximal dilation (reduced flow reserve) are detrimental over time. The molecular mechanisms responsible for these adaptations have been mostly studied in cell culture or *in vitro* experiments using isolated peripheral small arteries, while less data in the coronary vessels are available.

To orchestrate the acute reduction of the luminal diameter, VSMCs exposed to circumferential stretch undergo depolarization, leading to activation of L-type Ca^2+^ channel Ca_v_1.2 and subsequent myogenic vasoconstriction. Several mechanosensitive ion channels, including TRPC6, TRPM4 and KCNQ (Welsh et al., 2002; Earley et al., 2007; Zhong et al., 2010) as well as ciliary PC1 and PC2 (Sharif-Naeini et al., 2009) contribute to this stretch-induced depolarization. Moreover, ligand-independent activation of the mechanosensitive GPCR AT_1a_R has also been shown to induce myogenic vasoconstriction in response to elevated blood pressure in resistance vessels (Schleifenbaum et al., 2014). Additionally, hypertension-induced elevation of circumferential stretch results in activation of endothelial NOX and subsequent scavenging of NO by ROS (Spescha et al., 2014). In isolated ECs, it was demonstrated that this ROS production is initiated by stretch-induced activation of integrin α5β1, causing phosphorylation of JNK and p66^shc^, eventually activating NOX (Spescha et al., 2014). The impairment in NO bioavailability is aggravated by hypertensive agents such as salt, ET-1, angiotensin II, renin and vasopressin (Neglia et al., 2011), which further contributes to enhanced vasoconstriction and adds to the increased vascular resistance (Lassegue and Griendling, 2004; Neglia et al., 2011).

In hypertensive animal models, medial hypertrophic remodeling, associated with VSMC proliferation, has been shown to occur in small arteries whereas eutrophic remodeling, marked by VSMC reorientation and ECM deposition, was the most common form of remodeling in the more distal arterioles (Owens et al., 1988; Stacy and Prewitt, 1989). An explanation for the different types of inward remodeling in vascular segments of different sizes is not readily found, but may be due to a difference in sensitivity to mechanical stimuli along the vasculature with flow-induced responses affecting the vascular tone mainly in proximal vessels and myogenic influences in more distal vessels. The variations in sensitivity to mechanical stimuli are accompanied by vessel size-related variations in lumen/wall (*R*/*h*) ratio, and therefore wall stress (*σ*). In arterioles these variables are both much smaller than in, for instance, the aorta leading to a different optimal response for a given increase in blood pressure (*P*) (Feihl et al., 2006). However, the responses in the proximal and distal vasculature may also be related, in that an increase in peripheral vascular resistance in patients with hypertension can result in reduced shear stress in large arteries by inducing low peak systolic blood flow velocity and a low shear rate (Khder et al., 1998), thereby resulting in impaired shear-induced NO synthesis and progression of endothelial dysfunction.

Intriguingly, the exact nature of remodeling is also related to the pathologies underlying hypertension. Studies in small subcutaneous or omental vessels isolated from non-diabetic patients with essential hypertension indicated that these vessels undergo eutrophic remodeling (Schiffrin and Deng, 1996; Intengan et al., 1999; Schiffrin et al., 2000; Park and Schiffrin, 2001), whereas hypertension associated with diabetes or renovascular disease was shown to promote media hypertrophy in small arteries (Rizzoni et al., 1996; Schofield et al., 2002; Endemann et al., 2004). It could be speculated that the presence of co-morbidities such as diabetes and renovascular disease induces systemic low-grade inflammation and enhances oxidative stress, which in turn could directly interact with the outcome of mechanosignaling pathways. Lack of NO bioavailability, due to ROS scavenging in ECs, not only impairs NO-mediated NF-κB inactivation via S-nitrosylation (Kelleher et al., 2007), but also limits cGMP-mediated inhibition of VSMC proliferation. Wall stress-induced activation of NOX in ECs may subsequently result in ROS-mediated stimulation of AP-1 (Brandes et al., 2014). A similar activation of NF-κB and AP-1 has been observed in isolated coronary VSMCs in response to stretch (Hishikawa et al., 1997). Activation of these factors in both ECs and VSMCs, potentially coinciding with stretch-mediated nuclear accumulation of the focal adhesion protein Zyxin (Cattaruzza et al., 2004; Wojtowicz et al., 2010), results in transcription of inflammatory factors, including interleukin 6 (IL-6) and monocyte chemoattractant protein 1 (MCP-1), thereby stimulating monocyte extravasation (Zampetaki et al., 2005; Spescha et al., 2014). By secreting a variety of MMPs, these monocytes facilitate extracellular matrix reorganization and VSMC reorientation, thereby potentiating structural arterial remodeling (Intengan and Schiffrin, 2001; Wenzel, 2019). IL-6 also enhances VSMC motility (Wang and Newman, 2003), and like MCP-1, directly stimulates VSMC proliferation (Ikeda et al., 1991; Viedt et al., 2002). In addition, the stretch-activated Ca^2+^ channel Piezo1 also appears to be prominently involved in hypertension-dependent arterial remodeling. Piezo1 is highly expressed in VSMCs of murine small-diameter arteries (e.g., cutaneous caudal artery and cerebral arteries) and, while it is not involved in the myogenic response, VSMC-specific knockout attenuates hypertension-induced inward remodeling and VSMC hypertrophy in the cutaneous caudal resistance artery. The exact mechanism by which Piezo1 orchestrates arterial remodeling upon activation by wall stress is not completely understood, but Ca^2+^-dependent activation of TG2 may be involved (Retailleau et al., 2015).

A consistent finding in both clinical and experimental hypertension is capillary rarefaction, either structural (anatomic absence) or functional (non-perfusion), indicating that hypertension-induced vascular remodeling is not restricted to resistance arteries (Feihl et al., 2006). Experimental data in rat models of hypertension show reduced structural myocardial capillary density in adult hypertensive animals, but interestingly, this was age-dependent, as young animals showed normal capillary densities despite significantly hypertrophied ventricles (Tomanek et al., 1982). These animal findings are in agreement with data in young and adult patients with aortic stenosis-induced cardiac hypertrophy, also showing capillary rarefaction only at adult age (Rakusan et al., 1992). Interestingly, structural capillary rarefaction may be preceded by functional capillary rarefaction, as data in non-diabetic hypertensive patients show that functional capillary rarefaction parameters (percent capillary recruitment in nailfold skin) were lower in patients with modest hypertension and correlated with endothelial dysfunction, despite normal structural capillary density (Cheng et al., 2008). Functional capillary rarefaction may thus be the result of upstream arteriolar dysfunction resulting in perturbations in capillary perfusion.

Although the pathophysiology underlying the hypertension-induced rarefaction is still not fully understood, several mechanisms have been proposed, either related to loss of existing vasculature or insufficient growth of new vasculature (angiogenesis). In mesenteric and skeletal muscle microvasculature of spontaneously hypertensive rats, for instance, it has been shown that rarefaction induced by endothelial apoptosis was attenuated by systemic application of cell permeable superoxide scavengers Tempol and Tiron, indicating the involvement of excessive ROS formation (Kobayashi et al., 2005). Moreover, it has been demonstrated that low or absent mechanical shear, downstream of inward remodeled resistance arteries, could hamper KLF2-mediated inhibition of Smad2 phosphorylation, thereby enhancing endothelial to mesenchymal transition and subsequent vascular loss (Boon et al., 2007; Lee et al., 2017). Furthermore, altered shear could also lead to rarefaction via impaired NO synthesis, causing reduced NO-dependent production of vascular endothelial growth factor (VEGF) by VSMCs, which stimulates endothelial survival (Dulak et al., 2000). Besides its role in vasodilation and endothelial survival, NO stimulates endothelial proliferation and migration, important mediators of angiogenesis. This has been clearly illustrated in eNOS-deficient mice, in which impaired angiogenesis and arteriogenesis induced by hindlimb ischemia could be restored by adenoviral expression of constitutively active eNOS (Yu et al., 2005). Similarly, coronary occlusion in mongrel dogs triggered a myocardial ischemia-induced increase in capillary density, which could be blocked by L-NAME (Matsunaga et al., 2002). This study showed that, in the absence of NO, enhanced activity of MMP-2 and -9 results in degradation of plasminogen into the antiangiogenic factor angiostatin. With respect to hypertension-induced impairment of angiogenesis, there is also evidence that reduced numbers of circulating endothelial progenitor cells (EPCs) are involved (Hill et al., 2003; Imanishi et al., 2005). Although the cause of the reduced number of EPCs in hypertension is still unclear, the finding that lowering blood pressure by angiotensin receptor blockers (Bahlmann et al., 2005) or ACE inhibitors (Pirro et al., 2007) could restore their circulating numbers, in combination with their role in vascular repair, maintenance and angiogenesis, is consistent with the concept that shortage of these cells contributes to hypertension-induced rarefaction.

### Aging

Advanced age is another independent risk factor for development and progression of coronary artery disease, IHD and heart failure, contributing to morbidity and mortality worldwide (Dhingra and Vasan, 2012). Data from both patient and animal studies indicate that aging induces changes in the functional and structural properties of the vascular system (Paneni et al., 2017). Vascular ageing comprises different aspects of arterial wall injury accumulated over a long period of time due to factors such as oxidative stress, low-grade inflammation, and activation of the sympathetic nervous system, all resulting in increased vascular stiffness (Laurent and Boutouyrie, 2021). In large arteries, augmented arterial stiffness results in an increase in the velocity of the pressure wave, which together with a stiffer peripheral vasculature results in augmentation of the systolic blood pressure and increased cardiac afterload.

Arterial stiffening involves changes in extracellular components of the arterial wall due to proteolytic degradation of elastin fibers shifting the load towards stiffer collagen fibres (Duca et al., 2016). VSMCs also undergo age-dependent changes such as alterations in the activity of the contractile filaments as well as in the molecular signaling pathways regulating actin polymerization, both directly contributing to the vascular stiffening (Kajuluri et al., 2021). Furthermore, adhesion to the extracellular matrix is increased (Zhu et al., 2012).

Further data point towards aging-associated endothelial dysfunction, showing that aging induces flattening and enlargement of ECs as well as cytoskeleton alterations affecting mobility and proliferation (Yeh et al., 2000; Shi et al., 2004). In the endothelium of aging rats, changes in gap-junction distribution and connexin expression were seen, which resulted in dysfunctional and leaky vessels (Yeh et al., 2000). Moreover, reduced NO bioavailability was documented in aging animals (Csiszar et al., 2002; Leblanc et al., 2008, 2009; Kang et al., 2009, 2011), related to endothelial inflammation (Csiszar et al., 2004), similarly to data obtained in aging healthy subjects, which indicate impairments in both NO and prostanoid pathways (Singh et al., 2002). This was confirmed in the human coronary circulation, where arteriolar flow-mediated dilation was shown to evolve from prostacyclin in the young, to NO in adulthood and to H_2_O_2_ later in life and/or with onset of coronary artery disease (Beyer et al., 2017). Furthermore, blunted flow-mediated dilation occurs with age, as shown in both humans (Drexler et al., 1989; Zeiher et al., 1993) and animal models (Csiszar et al., 2002; Kang et al., 2009) and could be improved by antioxidant therapy (Kang et al., 2009).

Moreover, increased expression and elevated plasma levels of pro-inflammatory ET-1, as observed in ECs isolated from old vs young healthy subjects (Donato et al., 2009), were also associated with endothelial dysfunction, possibly via ROS-induced reduction in eNOS expression and activity (Wedgwood and Black, 2005). In 23 months old Fisher 344 rats, ROS production was shown to originate from NOX (Adler et al., 2003), presumably in response to age-related low-grade systemic inflammation. In addition, vascular oxidative stress can result from endothelial and VSMC mitochondrial dysfunction, the efficiency of the mitochondrial respiratory chain diminishing with age, causing electron leakage and subsequent release of ROS (Ungvari et al., 2007). Accumulating evidence furthermore suggests that Nrf2 is involved in the age-related high vascular ROS levels. Besides its role in flow-mediated signaling, Nrf2 is a redox sensitive transcription factor stimulating the transcription of a variety of genes involved in the antioxidant response. In the aged vasculature, however, there is a markedly lower expression and activation of Nrf2 (Ungvari et al., 2011a,b), possibly due to an age-related impairment of mechanosensitive activation of Nrf2 (Ungvari et al., 2019). However, this proposed aging-induced impairment of shear-mediated Nrf2 activation cannot explain why reporter studies in statically cultured VSMCs isolated from aged macaques demonstrate lower H_2_O_2_-induced transcriptional activity of Nrf2 when compared with the activity in cells isolated from young animals (Ungvari et al., 2011a). Regardless of what causes Nrf2 dysfunction in the aging vasculature, it limits oxidative stress resilience and stimulates NF-κB-mediated inflammation.

In addition to scavenging of NO by ROS, impaired eNOS activation has also been linked to ageing, as different studies have illustrated that shear-induced eNOS activation is blunted in the endothelium of aged animals (Sun et al., 2004; Yang et al., 2009). This could be mediated by lower availability of the eNOS substrate L-arginine and eNOS dimerizing co-factor tetrahydrobiopterin (BH4; Yang et al., 2009), but also by age-related ECM remodeling and subsequent stiffness. Aging is, as mentioned above, associated with increased ECM crosslinking, which, in combination with impaired elastin synthesis and enhanced elastin fragmentation and calcification, results in arterial stiffness. In cultured ECs, exposure to pulsatile flow leads to PI3K-mediated activation of Akt and subsequent eNOS activation in distensible, but not in stiff tubules (Peng et al., 2003). Similarly, exposure of ECs to laminar flow when cultured on hydrogels with mechanical properties resembling those of young blood vessels has been shown to induce higher NO production when compared to ECs cultured on stiff hydrogels (Kohn et al., 2015). Recent studies in human skeletal muscle biopsies have also demonstrated that a lower eNOS phosphorylation in aged tissue in response to acute passive leg movement-induced flow was accompanied by lower phosphorylation of PECAM-1, suggesting this endothelial mechanosensor may be involved in reduced shear-stress responsiveness (Gliemann et al., 2018).

Besides functional alterations, aging also promotes structural vascular changes in coronary resistance vessels (Hanna et al., 2014; Mccallinhart et al., 2018). Data from the Framingham heart study indicate that, with ageing, systolic blood pressure slowly increases due to increased peripheral vascular resistance and arterial stiffness (Franklin et al., 1997). Additionally, aging-induced blunting of beta adrenergic-mediated vasodilation, as well as activation of the RAAS system in the aged arterial wall may also contribute to the rise in systolic blood pressure in the ageing population (Jiang et al., 2008). Animal studies showed that aging results in increase in angiotensin II and aortic MMP-2 activity, inducing arterial remodeling (Wang et al., 2003, 2005). Moreover, as discussed earlier, the increase in blood pressure resulting from the increased vascular resistance induces arterial inward remodeling, promoting a further increase in blood pressure, forming a vicious circle.

Aging induces vascular alterations not only at the arterial level, but studies in dogs indicate that capillary density and length was also lower in the endocardium of old animals (Tomanek et al., 1991). This might be explained by the decreased angiogenic capacity of the aging heart as pathways central to vessel formation, such as hypoxia-inducible factor-1α (HIF-1α), PGC-1α, and eNOS, are affected by aging (Lahteenvuo and Rosenzweig, 2012), contributing to the mismatch between angiogenesis and cardiac hypertrophy in the aged heart. This impaired angiogenic response, besides the overall downregulation of angiogenic growth factors in aged tissue, seems to result from endothelial dysfunction and the associated low bioavailability of NO, which as described before, is an important mediator of angiogenesis (Matsunaga et al., 2002; Yu et al., 2005).

Intriguingly, vascular aging appears to progress differently in men and women (Dupont et al., 2019). The onset of menopause, which marks the end of a woman’s menstrual cycles, is associated with accelerated vascular aging, different from the gradual alterations in vascular function and structure as occur with chronological aging (Lakatta and Levy, 2003). The main mechanisms responsible relate to the hormonal changes inducing endothelial dysfunction, as it has been shown that reduced estrogen-mediated generation of NO likely underlies the progressive decline in endothelial function (Green et al., 2014). The reduced NO bioavailability is multifactorial, however, impaired availability of BH4 (Moreau et al., 2012), increased oxidative stress (Novella et al., 2012) and inflammation (Moreau et al., 2013) are likely contributors. Additionally, postmenopause has been associated with elevations in the vasoconstrictors ET-1 and norepinephrine (Green et al., 2014). Such imbalance between NO, ET-1 and norepinephrine contributes to the impaired vasodilation and sustained vasoconstriction observed in postmenopausal animal models (Knowlton and Lee, 2012), as well as to the increased arterial stiffness (Zaydun et al., 2006). Additionally, estrogen may not only enhance vascular relaxation via increased NO production, but may also promote angiogenesis. Estrogen receptor alpha gene knockout was associated with a decrease in VEGF levels and capillary rarefaction in the heart (Jesmin et al., 2010). In accordance with these findings, estrogen can inhibit TNFα-induced apoptosis by binding to its estrogen β-receptor, which induces Akt phosphorylation and Notch1 expression, thereby promoting vascular EC survival (Fortini et al., 2017). Altogether, these functional and structural alterations may explain, at least in part, the ischemic symptoms observed in postmenopausal women with chest pain, but no evidence of obstructive coronary artery disease.

### Chronic Coronary Artery Stenosis

Presence of a stenosis in an epicardial coronary artery has hemodynamic consequences for the distal vasculature. With severe stenosis, pressure and flow distal to the stenosis are compromised, resulting in impaired perfusion of the distal myocardium. An important clinical parameter used to define a stenosis as flow-limiting is the coronary pressure derived fractional flow reserve (FFR). This is the ratio between pressure distal and proximal to the stenosis during maximal coronary vasodilation, which is considered flow-limiting if below 0.8. Another parameter used for diagnostic and interventional purposes is CFR, which is defined as the maximum CBF, divided by the resting CBF and has a cut-off value of 2 (Stegehuis et al., 2018).

Due to the pressure drop across a flow-limiting stenosis, the perfusion pressure for the distal vasculature gradually decreases, lowering wall stress and thus the myogenic response due to blunted stimulation of stretch-activated mechanosensors (TRPs, KCNQ, etc.; Welsh et al., 2002; Earley et al., 2007; Zhong et al., 2010). The stenosis-induced impairment of perfusion and the resulting hypoxia may furthermore trigger red blood cell-dependent vasodilation via ATP release and subsequent activation of eNOS, S-nitrosohemoglobin-dependent bioactivity and NO synthesis via reduction of nitrite by deoxyhemoglobin (Kulandavelu et al., 2015). These autoregulatory mechanisms aim to lower vascular resistance in order to maintain flow and tissue oxygenation (Duncker et al., 2015). However, these mechanisms will be exhausted below perfusion pressures of about 40 mmHg, especially in the subendocardium, where the driving pressure is even lower due to the extra resistance of the transmural vessels and the increased extravascular compression, resulting in subendocardial ischemia.

Although microvascular dysfunction may be present already in the absence of a proximal coronary obstruction due to the presence of various comorbidities, hemodynamic changes induced by the stenosis in the distal vasculature can also directly cause alterations in both function and structure of the microvasculature, contributing to the reduced CFR in the myocardial area supplied by the stenotic artery. In a coronary stenosis model in dogs, a progressive increase in distal vascular resistance was documented in the first hours after the placement of the occluder, which was suggested to be mediated by withdrawal of the adenosine-induced vasodilation (Gorman et al., 1985). In a porcine model, 3 months after placing of an external occluder around the proximal LAD, increased vasoconstrictor response to ET-1 was present in subendocardial arterioles distal to the stenosis (Sorop et al., 2008). Vascular functional studies in chronically occluded hearts showed that endothelium-dependent relaxation in response to either bradykinin, substance P or adenosine diphosphate (ADP), was significantly impaired in arterioles isolated from the collateral-dependent region as compared with arterioles from the remote area in the same heart or compared with vessels from control, nonoccluded hearts (Sellke et al., 1992, 1996a,b; Park et al., 1996; Sodha et al., 2008). In contrast, reactivity to the endothelium-independent NO donor nitroprusside was not affected (Park et al., 1996; Griffin et al., 2001; Sodha et al., 2008). In Yucatan miniswine, 22 weeks after the placement of an ameroid occluder around the LCX, the bradykinin-mediated dilation in collateral-dependent arterioles was impaired, and could be improved by exercise-induced BK_Ca_-channel activation, suggesting a possible role for H_2_O_2_ (Xie et al., 2013). The vasoconstrictor response to ET-1 also appeared to be significantly increased in collateral-dependent arterioles or arterioles from the stenotic area, as compared to remote arterioles, a response that was due to a loss of ET_B_ receptor-mediated vasodilation (Park et al., 1996; Sorop et al., 2008). Combined, these data suggest that, even in absence of comorbidities, hemodynamic changes in the microvasculature distal to a coronary stenosis or occlusion impair NO bioavailability. Paradoxically, both total levels of eNOS, as well as phosphorylation of eNOS were shown to be increased in Yucatan miniswine arterioles distal to a coronary occluder (Xie et al., 2012). It is in that light, however, important to note that uncoupling of the eNOS dimer, due to oxidation of co-factor BH4, results in eNOS-mediated production of superoxide rather than NO (Channon, 2004).

The functional alterations distal to a coronary stenosis or occlusion were associated with structural changes in vessels of different sizes. Thus, in both swine and rats, the presence of a gradual proximal occlusion resulted in inward remodeling of the resistance arteries, either hypertrophic or eutrophic (Mills et al., 1994; Sorop et al., 2008; Canty and Suzuki, 2012). Additionally, microvascular (200–500 μm diameter) and capillary rarefaction was documented both in the subepicardium and the subendocardium (Urbieta Caceres et al., 2011), all these changes contributing to a reduced CFR and an increased minimal microvascular resistance in the myocardium distal to the stenosis. These data contrast with observations in patients with a coronary artery stenosis (Verhoeff et al., 2005) in which, upon revascularization, a maintained or even slightly reduced minimal microvascular resistance was observed. Such discrepancy may have several explanations. On the one side, vascular remodeling may have been influenced by the medication of the patients. Indeed, inward remodeling of isolated arterioles at low traluminal pressure, as present distal to a significant proximal stenosis was prevented by incubation with Ca^2+^ antagonist amlodipine (Sorop et al., 2006). On the other side, such changes in vascular diameter and density (arterial or capillary) may vary in time during the progression but also after the removal of the stenosis. A recent study by Weil et al., in swine, has shown that distal to a critical stenosis subendocardial arterioles show inward remodeling with increased arteriolar wall thickness and a reduction in lumen area. Interestingly, this was compensated by an increase in arteriolar as well as capillary density. However, 1 month after revascularization, such compensatory adaptation was lost, as subendocardial arteriolar and capillary density normalized, but the arterial inward remodeling persisted (Weil et al., 2020). These findings can explain the reduced subendocardial flow reserve late after revascularization despite a near normal vasodilator reserve immediately after PCI as seen in the study by Verhoeff et al. (2005). Furthermore, the persistent coronary microvascular inward remodeling could explain the blunted vasodilator response to dobutamine observed one month following revascularization, in swine, during increased myocardial oxygen demand (Kelly et al., 2011).

Vascular remodeling at the arteriolar level may be related to alterations in the microvascular (endothelial) function, such as the increased vasoconstrictor response to ET-1 as shown in the arterioles distal to a chronic LAD stenosis (Sorop et al., 2008), but altered mechanical factors may also contribute to this response. As such, altered extravascular compression by the dysfunctional hibernating or stunned myocardium (Canty and Fallavollita, 2005; Sorop et al., 2008; Canty and Suzuki, 2012) may result in abnormal mechanosensing. In addition, the blunted myogenic response as shown in sub-endocardial arterioles distal to the stenosis (Sorop et al., 2008) in conjunction with the reduced perfusion pressure and flow may promote inward remodeling, as seen in resistance arteries cultured at 40 mmHg (Sorop et al., 2006). Similarly, reduction of blood flow in mesenteric arteries by means of artery ligation resulted in eutrophic inward remodeling four weeks after surgery (Pourageaud and De Mey, 1997). This inward remodeling could partially be explained by observations in isolated ECs, demonstrating that low wall stress results in transcriptional activation of NF-κB (Pedrigi et al., 2017), which promotes vascular remodeling via monocyte recruitment and VSMC proliferation. Activation of NF-κB, in combination with poor perfusion-associated hypoxia and subsequent activation of HIF1α, furthermore stimulates transcription of ET-1 (Yamashita et al., 2001; Bourque et al., 2011; Stow et al., 2011). Experiments in a murine endothelium-specific ET-1 overexpression model illustrate that, besides its vasoconstrictor effects, ET-1 enhances the media to lumen ratio in mesenteric resistance vessels (Amiri et al., 2004). In cultured coronary artery VSMCs (Wu et al., 2007) and mesenteric arteries (Amiri et al., 2004), ET-1 was also shown to stimulate ROS production, which is prominently involved in remodeling of resistance arteries (Pires et al., 2010; Martinez-Lemus et al., 2011). The intermediate filament Vimentin, on the other hand, seems to prevent low flow-induced medial hypertrophy, as illustrated by a substantially increased wall thickness and VSMCs displaying a proliferative synthetic phenotype in ligated carotid arteries of Vimentin knock-out mice when compared with ligated carotids of wildtype mice (Schiffers et al., 2000; Van Engeland et al., 2019).

## Conclusions and Future Directions

The coronary microvasculature depends critically on acute as well as chronic adaptations of vascular diameter, density, and structural dimension to couple myocardial perfusion to myocardial oxygen demand. The effectiveness of these adaptations, however, is impeded by classic cardiovascular risk factors, including metabolic dysregulation, hypertension, aging and atherosclerosis. Accumulating evidence indicates that these risk factors modulate the interaction between the myriad of mechanisms regulating vascular function and structure and the many biomechanical cues, including shear stress, wall stress and stretch, and extravascular compressive forces, and the signal transduction pathways they induce.

Here, we discussed the involvement and consequences of biomechanical signal transduction cascades on functional and structural modifications of the coronary microvasculature and how they are influenced by the different risk factors (Figure 4). Although IHD patients often present with different combinations of these risk factors, and therefore most likely benefit from a tailored therapeutic approach focussed at normalization of hemodynamics (e.g., blood pressure) or restoration of mechanotransduction (e.g., glycocalyx in diabetes), it has become apparent that certain mechanistic and phenotypic aspects of coronary microvascular dysfunction overlap. Dysfunctional NO synthesis, as well as excessive ROS production, both linked to altered mechanosignaling (Figure 4), are consistently implicated in the pathophysiology of IHD and therefore, eNOS and NOX represent interesting therapeutic targets. Figure 4 furthermore illustrates that a reduction in microvascular density is a common feature of IHD in the presence of metabolic disorders and hypertension, as well as upon aging. Attenuating vascular rarefaction could therefore be a promising approach, in which novel insights in the behavior of pericytes, detachment of which from the microvasculature often precedes functional and structural vascular rarefaction (Kramann et al., 2017), may provide novel therapeutic leads. From studies in the field of oncology it has come forward, however, that especially for therapies aimed at modulating the microvasculature, perhaps even the angiogenic capacity, a tissue-specific approach is highly desirable, a challenge for which recent findings regarding the use of adeno-associated viruses in cardiac disease may provide a useful solution (Hinkel et al., 2017).

In order to fully understand the mechanisms underlying the alterations in coronary perfusion with the aim to improve the perspective of patients with IHD, it is essential that future studies acknowledge and deepen our understanding of the central role played by biomechanical signaling in the coronary microvasculature. In this regard, an integrative approach, taking both functional and structural modifications in consideration, will be essential.

## Author Contributions

MB and OS drafted the manuscript. DD, CC, and DM reviewed and approved the final version of the manuscript. All authors contributed to the article and approved the submitted version.

## Funding

We acknowledge the support from the Dutch CardioVascular Alliance: An initiative with support of the Dutch Heart Foundation (Grants 2017B018 ARENA-PRIME and 2020B008 RECONNEXT).

## Conflict of Interest

The authors declare that the research was conducted in the absence of any commercial or financial relationships that could be construed as a potential conflict of interest.

## Publisher’s Note

All claims expressed in this article are solely those of the authors and do not necessarily represent those of their affiliated organizations, or those of the publisher, the editors and the reviewers. Any product that may be evaluated in this article, or claim that may be made by its manufacturer, is not guaranteed or endorsed by the publisher.
